# Genetic mapping of developmental trajectories for complex traits and diseases

**DOI:** 10.1016/j.csbj.2021.05.055

**Published:** 2021-06-06

**Authors:** Eldad David Shulman, Ran Elkon

**Affiliations:** Department of Human Molecular Genetics and Biochemistry, Sackler School of Medicine, Tel Aviv University, Tel Aviv, Israel

**Keywords:** single-cell RNA sequencing, Genome-wide association studies, Computational methods, Genomics, Transcriptomics, Developmental biology

## Abstract

Genome-wide association studies (GWAS) have identified numerous common genetic variants associated with complex human traits and diseases. However, the translation of GWAS discoveries into biological and clinical insights is highly challenging. In this study, we present a novel bioinformatics approach for enhancing the functional interpretation of GWAS signals, based on their integration with single-cell (sc)RNA-seq datasets that examine developmental processes. Our approach performs three tasks: (1) Identification of links between cell differentiation trajectories and traits; (2) Elucidation of biological processes and molecular pathways that underlie such trajectory-trait links; and (3) Prioritization of target genes that carry the links between trajectories, pathways and traits. We applied our method to a set of 11 traits of various pathologies, and 12 scRNA-seq datasets of diverse developmental processes, and it readily detected well-established biological connections, including those between the maturation of cortical inhibitory interneurons and schizophrenia, hepatocytes and cholesterol levels, and pancreatic beta-islet cells and type-2 diabetes. For each of these associations, our method pinpointed top candidate genes that are strongly associated with both the kinetics of the differentiation trajectory and the disease’s genetic risk. By the identification of trajectory-disease links, molecular pathways that underlie them and prioritizing candidate risk genes, our method improves the understanding of the etiology of complex diseases, and thus holds promise for enhancing rational drug development that is aimed at targeting specific biological processes that mediate the genetic predisposition to diseases.

## Graphical abstract

**Figure:**
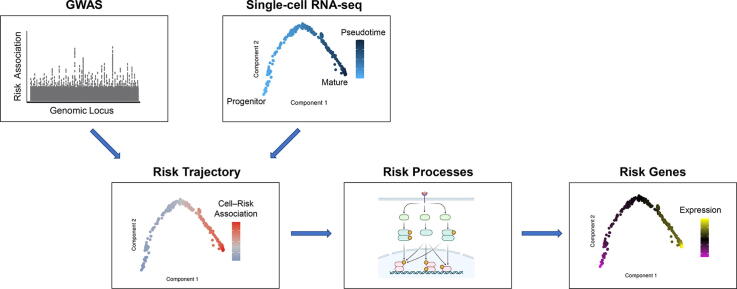

## 1 Introduction

Over more than a decade, genome-wide association studies (GWAS) are used to systematically identify common genetic variants, mostly single nucleotide polymorphisms (SNPs), that are associated with complex human traits and diseases [1]. At present (Feb. 2021), the GWAS catalog already documents more than 245 k associations from more than 4,800 studies [2]. These discoveries herald the initial fulfillment of the great expectations that were set with the completion of the Human Genome Project for the impact of genomics on the diagnosis, treatment, and prevention of complex diseases [3], such as cancer, heart diseases, mental illnesses and age-related maladies as Alzheimer and Parkinson, which are the leading cause of morbidity and mortality in the developed world. A prerequisite for translating GWAS discoveries into improved disease treatment and prevention is elucidation of the molecular mechanisms by which these risk variants and their target genes affect disease pathogenesis. However, the translation of GWAS discoveries into biological and clinical insights is a daunting task, which poses one of the major challenges of current human genetics research [1].

The great challenges in the functional interpretation of GWAS results stem from two main reasons. First, most common diseases are highly polygenic; that is, their genetic predisposition is affected by hundreds or even thousands of genetic variants, with each one individually exerting only a very weak effect [4]. Thus, understanding the impact of numerous parallel weak effects on the cell's and organ's functionality calls for network-based bioinformatics analyses [5]. Second, unlike mutations causing Mendelian diseases, which mainly disrupt protein-coding sequences, the vast majority (greater than 90%) of genetic variants that affect our susceptibility to common diseases map to the noncoding part of the human genome [6]. Multiple studies showed that these noncoding risk variants are mainly located within genomic regulatory elements that control the expression of their target genes [7]. Thus, disruption of enhancer and promoter activity, leading to mis-regulation of gene expression, emerges as a principal mode of action for risk variants discovered by GWAS.

One avenue where marked advances in functional interpretation of GWAS results are being made is the illumination of the pathogenesis' cellular architecture - that is, systematic identification of the specific organs and cell types in which the predisposing genetic variants exert their impact, as well as the genes and pathways that mediate this effect. This goal is bioinformatically pursued by integrated analyses of GWAS results and transcriptomic and epigenomics data, recorded over large panels of tissues and cell types. Following the derivation of cell-type- or tissue- specificity scores for gene expression levels or epigenetic signals, the next goal is to detect particular cell types/tissues that are significantly enriched for GWAS signals of the examined trait. While some studies restrict such integrative analyses to GWAS SNPs that passed a stringent genome-wide statistical significance threshold (e.g., [7]), many others apply methodologies that build on the fact that ample genetic information is also carried by a multitude of additional genetic variants that are truly associated with the examined trait, but fail reaching significance threshold due to their mild effect (and current GWAS sample sizes) [8]. Accordingly, such bioinformatics methods for integrative analysis of genetic and omics data, use GWAS summary statistics that record the association signals for all the SNPs (typically, in the order of a million) examined by GWAS. Prominent computational tools that implement such approaches include LDSC [9], MAGMA [10], and RolyPoly [11].

Most studies that carried out such integrated analyses used transcriptomic and epigenomics data obtained from bulk tissues. For example, introducing the LDSC-SEG approach, Finucane et al. integrated GWAS summary statistics for 48 diseases and traits and GTEx RNA-seq data from 53 tissues or cell types [12] and gene expression array data from 152 tissues and cell types [13], and recapitulated known biological connections, e.g., between Lupus and immune cells, type-2 diabetes and pancreas, and LDL and liver cells. However, the precision in determining specific cell types that can be obtained by bulk analysis of heterogeneous tissues is limited. Therefore, recent single-cell transcriptomic techniques (scRNA-seq), which have transformed the resolution by which the profile of distinct cell types that constitute composite organs can be determined [14], are now harnessed to this bioinformatics task of prioritizing cell types that are most relevant to the pathogenesis of complex diseases. For example, Skene et al. [15] sought brain cell types that underlie Schizophrenia (SCZ) etiology. To this goal, they calculated cell-type specificity scores per gene using single-cell transcriptomic datasets from human and murine brains, and SCZ-association gene scores using SCZ GWAS summary statistics. Given these two scores, they searched for cell types whose expression-specificity gene scores are highly correlated with SCZ-association gene scores. Notably, a clear connection with SCZ risk signal was detected for only 4 of the 24 main brain cell types (Medium spiny neurons, pyramidal cells in hippocampal CA1, pyramidal cells in the somatosensory cortex and cortical interneurons). Similarly, Watanabe et al. [16], adapting the popular MAGMA tool [10] for this task, analyzed 43 publicly available scRNA-seq datasets and GWAS summary statistics for 26 traits, and pinpointed highly specific cell types as connected with the etiology of the analyzed conditions.

Previous studies that integrated GWAS results and scRNA-seq data mostly considered cells as static entities. However, cells dynamically change their state, most drastically during differentiation. Recently, several algorithms were developed to infer, from single-cell transcriptomic data, the organization of cells by temporal or developmental stage in an abstract regulation space [14], [17], [18]. Prominent among them is Monocle, which reconstructs a trajectory along which the cells profiled in the experiment are presumed to travel during the differentiation process, and then projects each cell onto this trajectory at the proper position ('pseudotime'). Each cell's pseudotime value is measured as the distance along the trajectory from its position back to the track's start point. To describe complex differentiation processes in which cells make fate decisions, Monocle allows these trajectories to have a branched structure with multiple possible outcomes or “lineages” [19], [20].

In this study, we go beyond previous studies that sought connections between traits and cell types, to search for connections between traits and developmental trajectories. Analyzing GWAS summary statistics of 11 traits and diseases and 12 scRNA-seq datasets that examined differentiation of a multitude of organs, we pinpointed specific developmental trajectories that are linked to disease pathogenesis, and elucidated biological processes and target genes that underlie these connections.

## 2 Results

**Fig. 1:**
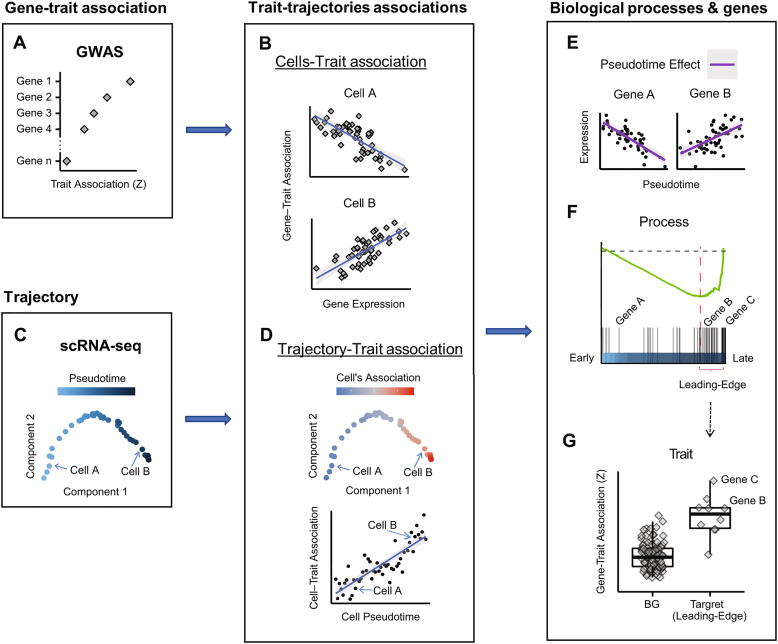
Our method for identification of links between developmental trajectories and traits, and biological processes and genes that underlie these links. A. Gene-trait association scores are derived from GWAS summary statistics. B. Each cell from the scRNA-seq dataset is assigned a cell-trait association score, based on the correlation between its gene expression profile and gene-trait association scores. Cells A and B exemplify cells with low and high cell-trait association scores, respectively. In Cell B, which is located towards the end of the trajectory, trait-associated genes tend to be highly expressed. C. Trajectory analysis of scRNA-seq datasets is used to infer individual cells' maturation states (pseudotime). D. Correlation between cells' pseudotime and cell-trait association scores indicate a link between the differentiation trajectory and the trait. In trajectories linked to the trait, cell-trait association scores increase along pseudotime. E. To identify biological processes that underlie the association between trajectory and trait, we first identified, using GSEA, gene sets associated with the trajectory pseudotime. For this analysis, the pseudotime effect on each gene (that is, the incremental change in expression along the trajectory) was inferred. F. Pseudotime effects were used to rank the genes in a list, such that genes whose expression decreases with pseudotime (Gene A in the illustration) are at the beginning of the list, and genes whose expression increases (Gene B) are at the end of the list. GSEA is used to identify gene sets whose members are overrepresented at the end of the list. G. The leading-edge subset (defined by GSEA) of each gene set that is linked with the trajectory, is examined for association with the trait. Gene B and C are members of a gene set that is both linked to the trajectory and associated with the trait. Moreover, these genes themselves are both strongly associated with the trait and highly induced along the trajectory (F), suggesting that they contribute to the connection between the trait, trajectory, and biological process.

In this study, we implemented a novel bioinformatics approach for identifying associations between complex human traits and developmental trajectories, and biological processes and genes that underlie these associations. Our approach is based on an integrated analysis of GWAS summary statistics and scRNA-seq data, and performs three main tasks: **Task 1**. Identification of connections between developmental trajectories and traits. In this task we seek trait-trajectory links by identifying trajectories in which the transcriptomic programs of the differentiating cells shift towards expressing genes that are associated with the trait. To this goal, we first convert GWAS variant scores into ***gene-trait association scores*** (Fig. 1A). This is done using a statistical test implemented by MAGMA [10], which considers, per gene, the scores of the SNPs that overlap the gene body or its flanks (10 kbp), while controlling for the correlations between SNPs due to linkage disequilibrium (LD) patterns. Second, considering a scRNA-seq dataset, we calculate, per individual cell, a ***cell-trait association score***, which measures, using a regression model, the cell's propensity to express trait-associated genes (Fig. 1B, Methods). Focusing our analysis on scRNA-seq datasets that examined differentiation processes, we next used trajectory analysis to infer developmental trajectories in each dataset, and assign each cell with a pseudotime that reflects its differentiation state along a maturation pathway (Fig. 1C). Last, to identify differentiation trajectories that are linked to the analyzed trait, we examined the association between the cells' pseudotime and the cell-trait association scores (Fig. 1D). **Task 2**. Elucidate molecular pathways that underlie trajectory-trait links. This is done by searching for biological processes, whose genes, as a set, are both linked to the trajectory pseudotime and enriched for trait-association signals. In this task, to identify processes that are linked to the trajectory, we scored genes by the correlation between their expression and pseudotime (Fig. 1E), and then used GSEA tests [21] to identify functionally annotated gene-sets that are significantly associated with the trajectory's kinetics (Fig. 1F). Then, considering the leading edge of gene sets that passed the GSEA test (that is, the subset of genes in the gene set that carry the link with pseudotime), we used MAGMA's gene-set analysis to test for enrichment of this subset for the trait's GWAS signals (Fig. 1G). **Task 3.** Prioritize genes that contribute to the link between the pathway, trait and trajectory. In this final step, we examined the genes contained in the core enrichment of the gene sets detected in Task 2 and prioritize them according to their trait-association scores.

**Fig. 2:**
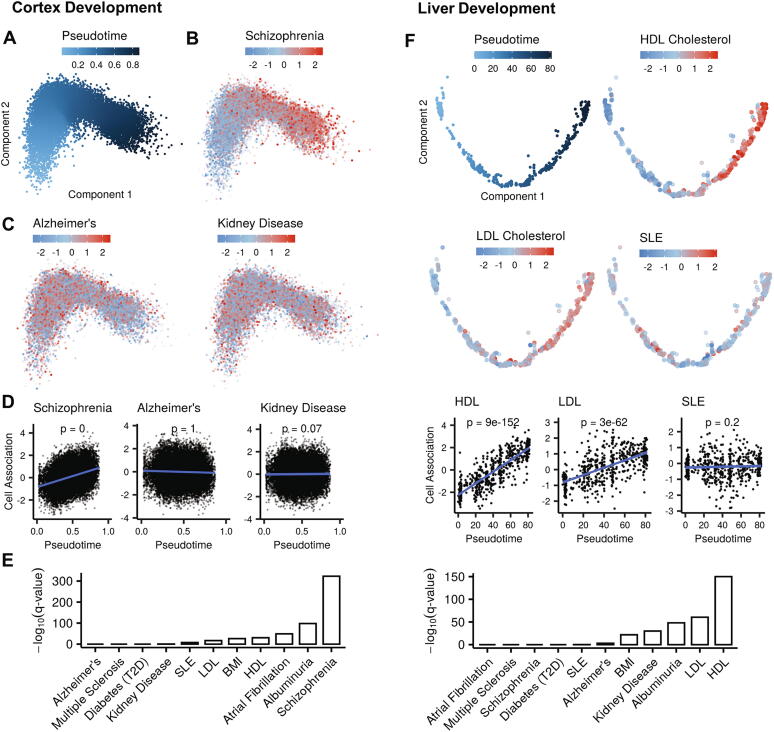
Developmental trajectories associated with traits. A. Pseudotime analysis of scRNA-seq of cells from the ganglionic eminences of embryonic mice (E13.5-E14.5), ordered the cells along a maturation trajectory of cortical inhibitory interneurons. Cells are colored according to their assigned pseudotime. B. The trajectory from A, with cells colored according to their association with schizophrenia risk (cell-trait association score). C. The trajectory from A, with cells colored according to their association scores with Alzheimer's (left) and chronic kidney disease (right). D. Regression analysis shows that cell-trait scores for schizophrenia significantly increase along the trajectory. In contrast, cell-trait scores for Alzheimer's and chronic kidney disease do not show such pattern. E. Bar plot showing the significance level (log10) of the association between the inhibitory-interneurons differentiation trajectory and multiple traits. The association of this developmental trajectory with schizophrenia is by far the most significant. F. Maturation trajectory in which hepatoblasts differentiate into liver hepatocytes (mice E7.5 - E15.5). Panels are as in A-E. This trajectory was highly associated with HDL and LDL levels.

We applied our bioinformatics method on a set of 11 traits of various pathologies, and 12 scRNA-seq datasets that examined various differentiation processes (Table S1). We first focused on the developmental scRNA-seq datasets in which pseudotime analysis delineated a single unbranched differentiation trajectory. As a first showcase, we present the analysis of a scRNA-seq dataset that probed cortical ganglionic eminence of embryonic mice (ages E13.5-E14.5) [22]. Pseudotime analysis of this dataset (including 9,669 cells) delineated a single maturation trajectory of cortical inhibitory interneurons (Fig. 2A). Notably, our method discovered that this trajectory is strongly associated with schizophrenia (Fig. 2B). Such association was not detected, for example, for Alzheimer's or kidney disease (Fig. 2C, D). Examination of all 11 traits included in our analysis showed that schizophrenia was, by far, the trait most significantly associated with this trajectory (Fig. 2E, Table S2), in line with recent evidence linking this trajectory to the pathogenesis of schizophrenia [23].

Our second showcase analysis of an unbranched differentiation trajectory was done on a scRNA-seq dataset that examined liver development [24]. Pseudotime analysis ordered the hepatoblasts, hepatocytes, and cholangiocytes cells (a total of 563 cells), extracted from mouse embryos (E10.5 to E17.5), along a developmental trajectory in which hepatoblasts differentiate into hepatocytes. Our method found that this trajectory is significantly associated with high density lipoprotein (HDL) and low density lipoprotein (LDL) cholesterol levels (Fig. 2F), two prominent traits controlled by the liver. Similarly, our method detected biologically highly relevant trait-trajectory connections for the other unbranched differentiation trajectories we analyzed, including links between Alzheimer's disease and microglia development [25] (Fig. S2A), adipogenesis [26] and extreme BMI (Fig. S2B), and kidney nephrons development [27] and kidney disease (Fig. S2C).

As cells progress through differentiation, they typically undergo substantial alterations in their identities driven by massive changes in cellular transcriptional programs. The above showcase analyses investigated for such examples of differentiation in the brain, liver, and adipose tissue. We next turned to examine if our methodology could be applied to more subtle cellular processes that are also associated with a genetic predisposition to complex diseases. As a test case, we analyzed a scRNA-seq dataset that profiled microglia cells upon viral infection [28]. Our method finds that this cell activation trajectory is strongly associated with multiple sclerosis (Fig. S2D) (This is in contrast to the link we detected between microglia development and Alzheimer's (Fig. S2A)). Our results are in line with a recent study that indicated that microglia affect the risk for multiple sclerosis via autoimmune processes in the central nervous system [29].

**Fig. 3:**
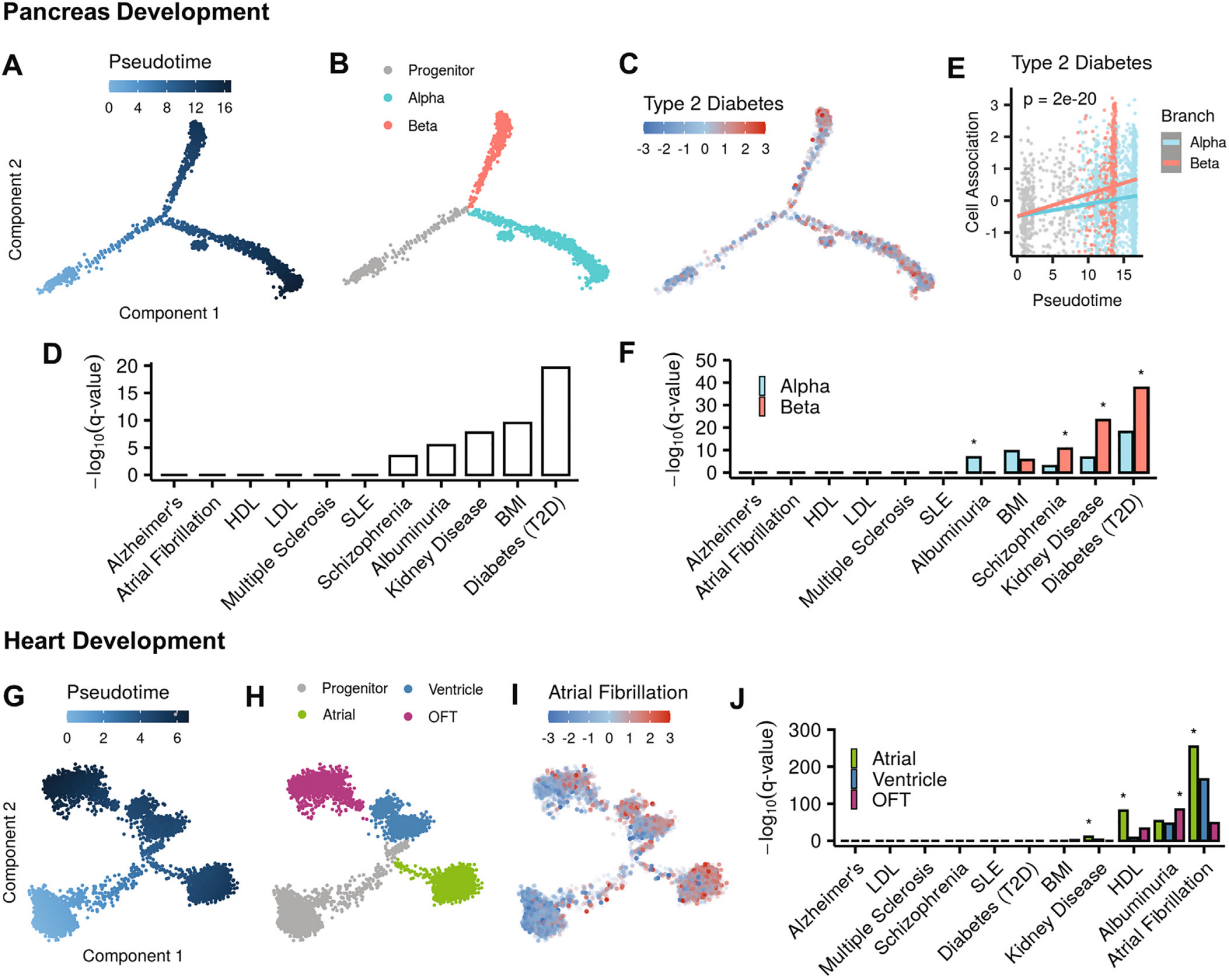
Associations between human traits and specific branches of developmental trajectories. A-B. Analysis of pancreatic cells from embryonic mice (E14.5) depicts the main developmental branching point, where progenitors make the decision towards alpha- or beta- islet cell fates. C. Cells are colored according to their association with risk for type-2 diabetes. D. Bar plots showing association of the pancreas trajectory with each trait. Diabetes is the most strongly associated trait. E. The risk for type-2 diabetes shows significant branch dependency (p-value for branch dependency was calculated using likelihood ratio test, see Methods). F. Bar plot showing trait association for each branch. Asterisks indicate that the association is significantly branch dependent (FDR q-value of likelihood ratio test < 0.05). G. A developmental trajectory of myocardium cells from the cardiogenic regions of mice embryos (E7.75, E8.25, E9.25). H. Pseudotime analysis delineates a three-branch trajectory. These branches terminate in cells that populate different anatomical areas within the heart: the atria, ventricles, and outflow tract (OFT). I. Cells are colored according to atrial fibrillation risk score. J. Association with risk for atrial fibrillation is significantly stronger in the atrial branch.

Next, we moved to analyze scRNA-seq datasets that showed branched differentiation trajectories; that is, developmental trajectories that include a single or multiple branching points, where cells that followed the same route continue their development along mutually exclusive paths, leading to distinct terminal states (Fig. S1A). A canonical branched differentiation trajectory is the one of the pancreatic islet cells development, where after the branching point, progenitor cells differentiate mainly to alpha or beta islet cells. We analyzed two scRNA-seq datasets that explored this differentiation process in embryonic mice at E14.5 [30] (Fig. 3A-B), and at E13.5-E15.5 [31] (Fig. S3A-B). As expected, analysis of both datasets showed that this trajectory, considered as a whole, was most strongly associated with type-2 diabetes (T2D) (Fig. 3C-D, Fig. S3C-D). Further examination demonstrated that association with T2D shows branch dependency, being significantly more pronounced for the trajectory that leads to the formation of beta cells (Fig. 3E, Fig. 3SE), in line with the well-established role for beta cells dysfunction in the pathophysiology of T2D [32]. Reassuringly, in both datasets, T2D was the trait most strongly associated with the beta-cells trajectory (Fig. 3F, Fig. 3SF). Additional single-branched trajectories that we analyzed included differentiation of neuronal progenitors from the cortical lob and pons of human embryos and fetuses [33] and maturation of blood B cells [34]. In the neuronal differentiation trajectory, progenitors develop into either intermediate progenitor cells (IPC), or radial glial (RG) cells (Fig. S3G). The top scoring trait associated with this trajectory was schizophrenia, and this link was significantly stronger with the RG branch (Fig. S3H-I). In the B cell maturation trajectory, we detected a strong association between systemic lupus erythematosus (SLE) and multiple sclerosis and the branch terminating in native and memory B cells (Fig. S3J-K).

Next, we moved to analyze differentiation trajectories that contain more than one branching point. As a first example, the differentiation trajectory of myocardium cells from the cardiogenic regions of mice embryos (E7.75, E8.25, E9.25) contains two branching points (Fig. 3G). The first branching point splits the progenitor cells into those that develop to atrial cells and those that continue to develop along a second path containing another branching point where cells follow tracks leading to the formation of either ventricles or outflow tract (OFT) cells (Fig. 3H). Our analysis found that atrial fibrillation is most strongly connected with the atrial branch (Fig. 3I-J). A similar branching pattern is displayed by a developmental process in the human fetal kidney. The first branching point splits the cells into those that proceed along a path which terminates in the formation of distal tubule/loop of Henle cells (DTLH), and cells that continue towards a second fate-decision point. This second branching leads to the formation of either early proximal tubule (ErPrT) or podocyte (Pod) cells (Fig. S3L). Notably, the top scoring trait associated with these differentiation trajectories was Albuminuria, which is a kidney malady. It was significantly associated with all the three branches, with strongest link to the one that leads to Pod cells (Fig. S3M). This developmental process was also linked to HDL levels, that showed an association specifically with the Pod branch (Fig. S3M).

**Fig. 4:**
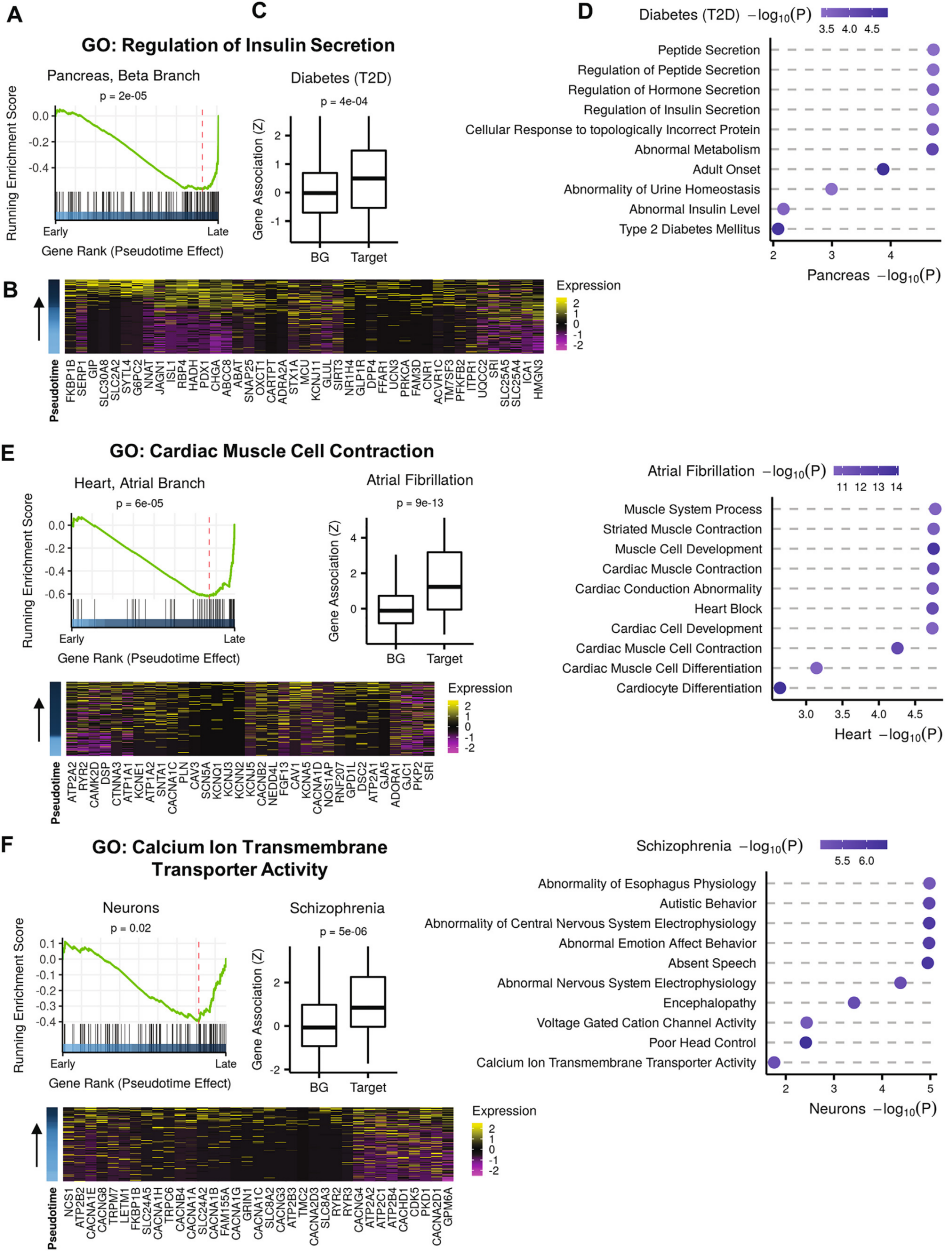
Characterization of molecular pathways underlying trajectory-trait associations. A. GSEA was applied to a list of genes ranked according to the incremental change of their expression along the branch of beta-islet cells in the pancreas developmental trajectory. The GO gene set ‘Regulation of Insulin Secretion’ was significantly over-represented at the end of the list (corresponding to genes whose expression is induced towards the end of the trajectory). Genes to the right of the dashed vertical red line (43 genes) constitute the leading-edge subset of the 'Regulation of Insulin Secretion’ gene set. B. A Heatmap of the leading-edge genes. Rows correspond to cells, arranged according to pseudotime (bottom to top), and columns correspond to genes. Color indicates scaled normalized expression levels. C. The subset of 43 leading-edge genes was associated with risk for type 2 diabetes. The boxplot shows the distribution of gene-T2D association scores for the leading edge (Target) and all other genes (BG); p-value calculated using MAGMA’s gene-set analysis. D. Biological processes (GO terms) that were associated with both the beta cells branch and type-2 diabetes. Color indicates the significance of the trait association. Shown are the ten gene sets whose leading-edge genes were most strongly associated with T2D. The same analysis was carried to the atrial branch – atrial fibrillation association (E) and the neurons progenitors – schizophrenia association (F). (For interpretation of the references to color in this figure legend, the reader is referred to the web version of this article.)

The analyses described above implemented the first task of our approach - identification of connections between developmental trajectories and traits. Following the identification of such links, we performed the second task – elucidation of molecular pathways and biological processes that underlie the trajectory-trait connections. To this end, we first score genes by the incremental change in their expression along the trajectory (Methods), use these scores to sort the genes, and then apply GSEA tests to identify functionally annotated gene sets that are significantly associated with the trajectory's kinetics. (GSEA tests seek gene sets whose genes are significantly over-represented towards the end of the sorted gene list). Following this method, GSEA analysis of the beta-islet cells' branch in the pancreatic dataset (E13.5–15.5) revealed enrichment for 'regulation of insulin secretion', pinpointing this process as a main biological end-point that is executed by this cell fate (Fig. 4A). The leading-edge genes, defined by GSEA for the 'regulation of insulin secretion' gene set, contains a subset of 43 genes that carry the link between this biological process and this developmental trajectory (Fig. 4B). Importantly, this subset of genes is also significantly associated with T2D (Fig. 4C). Thus, collectively, this analysis defines a core set of genes that function in secretion of insulin, are up-regulated specifically along the beta-cell trajectory and are associated with T2D risk. We identified additional gene sets, including 'abnormal insulin levels' and 'regulation of hormone secretion', which were both linked to the beta-cells trajectory and enriched for T2D risk signal (Fig. 4D, Table S3). Applying this analysis to the connection detected in the heart development dataset between atrial fibrillation and the atrial differentiation branch, pinpointed numerous gene sets, including 'cardiac muscle contraction' and 'cardiocyte differentiation' (Fig. 4E, Table S3). Similarly, we detected the biological processes 'calcium ion transmembrane transporter activity' and 'abnormal nervous system electrophysiology' as molecular pathways that underlie the connection between the radial glial differentiation trajectory and schizophrenia (Fig. 4F, Table S3). Results on molecular pathways and biological processes that underlie trajectory-trait connections detected in the other datasets we analyzed are shown in Fig. S4. In addition to detecting (without using any a priori biological knowledge) well-established links between traits, developmental trajectories and biological processes, our results also point to novel connections, such as possible interplays between kidney development and lipoprotein traits (nephrogenesis and LDL Fig. S2C; PTA cells development into podocytes and HDL Fig. S3M). The link between kidney function and lipoprotein levels has been previously speculated [35]. Interestingly, our analysis suggests that liver and kidney developmental trajectories may impact cholesterol levels through different biological processes (Fig S5, Table S3).

**Fig. 5:**
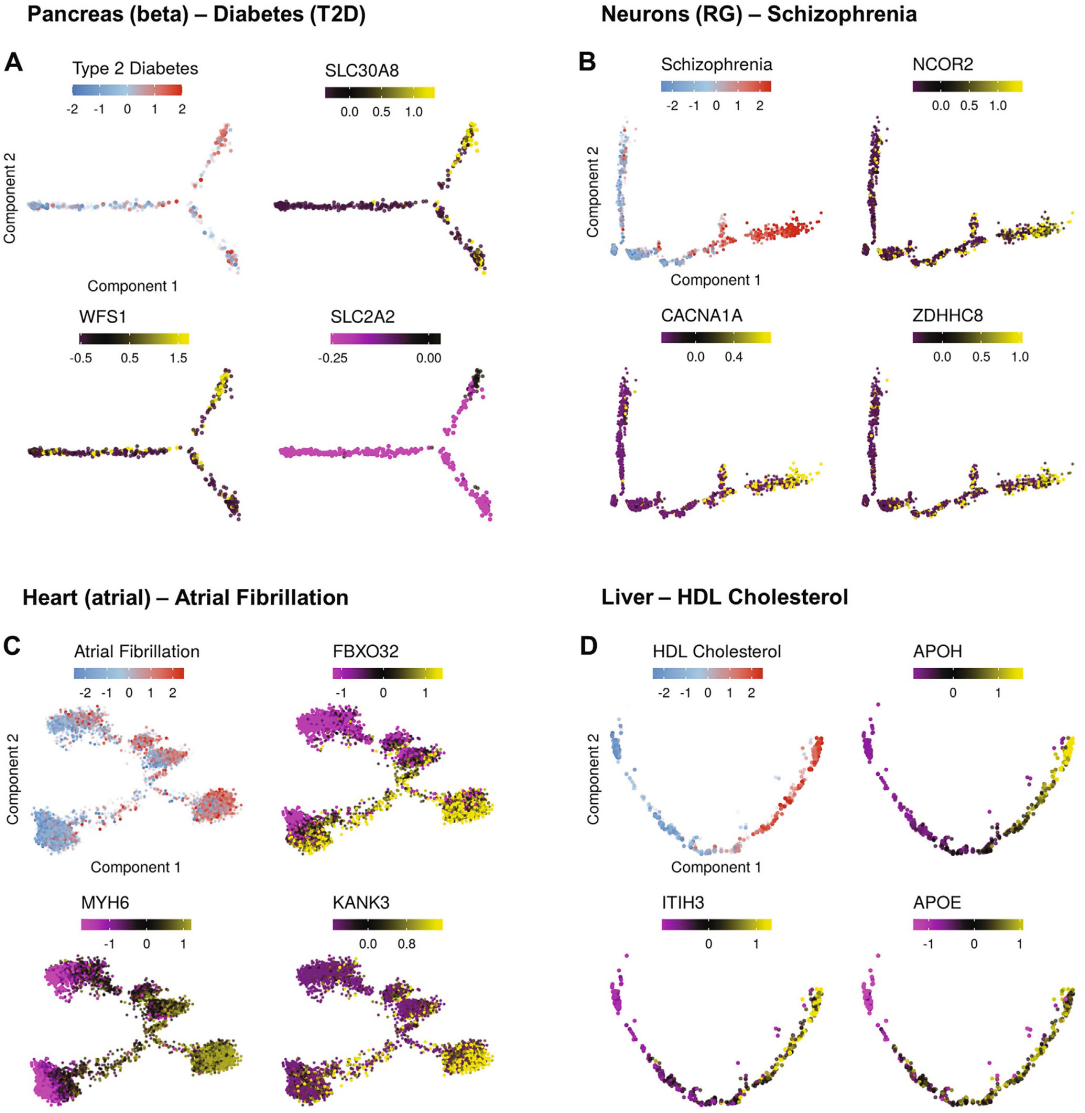
Candidate genes affecting the trajectory-trait associations. Shown are examples of genes that are included in the GSEA leading-edge subset of biological processes that are associated with the trajectory kinetics and are themselves enriched for trait-association signal. Expression levels are normalized and scaled. Shown are selected top candidate genes for the link between (A) beta cells trajectory and type 2 diabetes; (B) radial glial branch and schizophrenia, (C) heart development and atrial fibrillation and (D) liver differentiation and HDL levels.

The last task in our analysis pipeline is aimed at prioritizing genes that carry the links we revealed between molecular pathways, traits and trajectories. This is done by pinpointing genes contained in the leading edge detected by GSEA in task 2 (which indicate that their expression is induced along the trajectory) and are themselves significantly associated with the trait (according to their gene-trait score). As few examples, this analysis singled out 104 genes that are likely playing a key role in linking risk for T2D and the differentiation of beta cells (Table S4), among them SLC30A8 [36], [37], [38], [39], WFS1 [40] and SLC2A2 (GLUT2) [41] that were previously implicated as T2D risk genes (Fig. 5A). Among the top candidate genes pinpointed by the analysis of the link between neuronal RG differentiation and schizophrenia are ZDHHC8 [42], NCOR2 [43] and CACNA1A [44], which were previously implicated as SCZ risk genes (Fig. 5B). Analysis of the link between atrial cell differentiation and atrial fibrillation singled out known risk genes, such as MYH6 [45] and FBXO32 [46] (Fig. 5C). Top candidate genes identified for the liver development – HDL link include APOH [47], ITIH3 [48], and APOE [49] (Fig. 5D). Candidate genes detected in the other datasets analyzed in our study are shown in Fig. S6.

## 3 Discussion

In this study we presented a three-tier bioinformatics approach for identifying connections between developmental trajectories and genetic predisposition for complex traits, and characterizing molecular pathways and prioritizing candidate genes that underlie these links. Our method is based on an integrative analysis of scRNA-seq data exploring differentiation processes and GWAS summary statistics. Applying it to a set of 11 GWAS datasets and 12 scRNA-seq datasets, we demonstrated its capacity to detect well-established associations, such as maturation of hepatocytes and HDL and LDL levels; inhibitory neurons and schizophrenia; and pancreatic beta-islet cells and type-2 diabetes. In the second tier, our approach detects specific gene sets, each representing a particular biological process or molecular pathway, which underlie the identified trait-trajectory links. In the last tier, our method pinpoints highly scoring genes from the gene sets detected in the 2nd tier, prioritizing them for further examination.

Our approach has several limitations. First, the step in task 1 to derive gene-level risk scores based on SNP scores requires SNP-to-gene maps. Mapping SNPs to their genuine target genes is a key genomic challenge, considering the fact that risk variants are enriched at enhancer regions and that enhancers frequently control distal genes rather than the closest ones [50], [51], [52]. Given that the task of improving SNP-to-gene mapping is still under intensive investigation, which is out of the scope of our current study, in our analyses we opted to use MAGMA's default mapping (gene bodies + 10 kbp flanks). This suboptimal SNP-to-gene mapping neglects the impact of many distal risk SNPs and is occasionally expected to suffer from mapping of SNPs to the wrong target genes (e.g., in case an intronic risk SNP is located within an enhancer that regulates a gene different from its host gene).

A second limitation of our approach is that it seeks differentiation trajectories that are positively correlated with the trait (that is, the expression level of risk genes tends to increase along the trajectory). We focused on positive correlation as we found that it is the most common pattern. The adaptation of our method to cases of negative correlation (that is, cases in which the activity of disease-related molecular pathways decrease along the developmental trajectory) is straightforward. More challenging are cases in which the expression of genes associated with the trait shows a temporal non-monotonous pattern, which peaks at a certain intermediate cell state along the trajectory. For example, Cuomo et al. recently showed that during the induced differentiation of pluripotent stem cells (iPSCs), the impact of specific SNPs on the expression of their target genes is strongest in particular intermediate states [53]. To detect such trajectory-trait links, the linear model for inference of cell-trait associations used in our method (task 1; Fig. 1D) should be replaced by a more flexible one, such as natural splines regression. In most of the datasets analyzed in our study, the trajectory-trait links were modeled well by the linear framework, and no benefit was obtained by using the more flexible spline-based model (Fig. S7A-D). An exceptional case was the link between B cell maturation and SLE, where the strongest cell-trait association scores were obtained in an intermediate state (Fig. S7F), and thus it was modeled better by spline. To properly handle such non-linear cases, the method we apply in the second task - inferring biological processes linked with the trajectory (Fig. 1E-F) - should be adjusted as well. By default, for the GSEA tests we rank the genes according to the correlation of their expression with pseudotime. To account for non-linear associations between a trait and a trajectory, genes can be ranked by the fit of their expression pattern to the spline curve of the trait-trajectory association (Fig. S7G-H, Tables S3-4).

Current scRNA-seq data are characterized by high prevalence of 'drop-outs', referring to the fact that in each individual cell, only a subset of the expressed genes (typically 10–20%) is detected (also referred to as 'zero-inflation')[54]. To overcome this suboptimal measurement, previous studies that integrated scRNA-seq and GWAS data relied on averaging gene expression levels over multiple cells. Specifically, considering cell states as a static property and aiming to identify specific cell types that are enriched for trait-association signals [12], [13], previous methods are based on an initial definition of the cell types detected in a dataset (e.g., by clustering), followed by the calculation of expression level of each gene in each cell type (by averaging its expression overall individual cells assigned to each cell type). In contrast, our method considers the expression levels detected in each individual cell. Our results demonstrate that despite immense drop-out, individual cell measurements allow robust calculation of (individual) cell-trait association scores, enabling reliable detection of trajectory-trait connections. In the analyses presented in this study, for each dataset, we considered all genes that were detected in at least 10 cells. Of note, our results were grossly non-sensitive to the choice of filtering cutoff (Fig. S8). However, selecting genes based on variability of expression across cells, which is a common selection practice for cell clustering in single-cell analyses (highly variable genes), showed a much greater impact on the results (Fig. S8).

Our approach relies on a proper inference of the topology of the trajectory (e.g., correctly identifying the branching points along a trajectory), which often requires prior knowledge of the underlying biology for choosing the appropriate method and its parameters [55]. Therefore, in this study, we opted to follow, in all datasets, the trajectory inference method that was used in the original publications. Yet, our approach can be applied using many different trajectory inference tools that capture the topology of the dataset considered. Applying six different computational methods, we found that our results were robust to the choice of the trajectory algorithms (Fig. S9).

scRNA-seq data are accumulating at an unprecedented pace. As more and more differentiation processes are explored, our method will be able to reveal novel links between specific fate-determination trajectories and complex diseases and elucidate molecular pathways that are related to the disease etiology. By prioritizing top-scoring candidate genes, such analyses hold promise for enhancing rational drug development that is aimed at targeting biological processes that mediate the genetic predisposition to developing the disease. Furthermore, from this perspective, detecting an association between a disease and a dynamic trajectory has a conceptual advantage over an association with a static cell cluster: there is an “opportunity window” for preventive and therapeutic interventions to be effective. The kinetic pattern of the disease-trajectory associations can guide us to better definitions of such opportunity intervals for different diseases.

## 4 Methods

### 4.1 Analyzed datasets

We analyzed 12 publicly available scRNA-seq datasets that examined differentiation processes in different tissues in humans and mouse, and GWAS data of 11 human complex traits (Table S1).

### 4.2 scRNA-seq trajectory analysis

For each scRNA-seq dataset, we performed a pseudotime analysis (Fig. 1C) according to the procedure described in the original publication (we followed the code, which was either published or obtained from the authors). In the adipogenesis dataset (Fig. S2B), pseudotime was not inferred in the original publication. Therefore we used Monocle 2 [56], setting dimensional reduction to two components, and used the highly variable genes called by the original publication as the ordering genes.

### 4.3 Identification of associations between traits and trajectories

***Gene-trait association scores***. Gene-trait association scores were calculated using MAGMA's (v1.07) gene analysis [10] (Fig. 1A). This analysis takes as input GWAS summary statistics and a reference file to estimate LD between SNPs, which was inferred from the European samples in the 1000 Genome project [57]. Following MAGMA's default setting, for each gene, we considered the SNPs that either overlap the gene body or located within its 10 kbp flanking region.

***Cell-trait association scores***. For mouse datasets, we converted mouse genes to their human orthologues using biomaRt [58] and kept only genes with one-to-one Hs-Mm ortholog mapping. We excluded genes that were expressed in<10 cells. Gene counts were normalized using Seurat v3 [59] default normalization (log normalization). This normalization divides gene counts for each cell by the total counts for that cell, multiplies by 10,000, and last, values are transformed using a natural log (adding a pseudo count of 1).

To assign trait-association scores for each individual cell (Fig. 1B), we used MAGMA's gene property analysis to fit the following regression model to each cell:

$$Z=\beta_{0}+C\beta_{c}+A\beta_{A}+B\beta_{B}+∊$$

where Z is the vector of the genes' GWAS Z-scores converted from the p-values obtained from MAGMA's gene analysis for the trait. B is a matrix of technical confounders, including gene length and SNPs' LDs, calculated by MAGMA. C is the vector of normalized expression of the genes in the cell, and A is a vector of the average normalized expression for the genes in the dataset. The t-statistic of $\beta_{c}$ was taken as the score for the association between the cell and trait. This regression model tests for correlation between gene association with the analyzed trait and the gene expression profile of the analyzed cell. It is similar to that used by Watanabe et al. [16]; however, our regression was applied to expression levels from individual cells rather than the average expression levels of cell types.

***Association between cells' pseudotime and trait-association scores***. We examined the correlation between cell-trait association scores and the pseudotime assigned to the cells (Fig. 1D). For single branch trajectories, we fitted the following linear regression model:

Cell-Traitscore~Pseudotime

We performed a one-sided test (Pseudotimecoefficient>0). Significance indicates that cells' association with the trait increases along the trajectory's pseudotime. For trajectories with more than one branch (pancreas, neurons, B cells, kidney PTA, and heart datasets), to examine if the trait's association was branch-dependent, we used a procedure similar to Monocle's method for identifying branch-dependent genes (Census) [20]. Briefly, we compared the goodness of fit of the following two linear regression models:

Cell-Traitscore~Pseudotime

Cell-Traitscore~Pseudotime+Branch:Pseudotime

where the first model assumes that the trait is not branch dependent, and the second model, which contains the interaction term between branch and pseudotime, assumes branch dependency (Fig. S1). Likelihood ratio test was carried out using the R package VGAM [60] (*lrtest* function). As this approach requires the assignment of branch identity to each cell, including the cells before the branch point, we followed Monocle's approach: We divided the unbranched progenitors into branches by ordering them according to pseudotime, and alternatingly assigning odd and even ranked cells to the first and second branches, respectively. We assigned the first progenitor to both branches. (Similarly, in the case of three branches, progenitors were split according to ranking, assigning cells with rankings that follow the arithmetic sequences 1+3n,2+3n,3+3n (n = 0,1,2,3, …) to the first, second, and third branches, respectively).

Furthermore, to examine if there is a positive correlation between cell-trait association scores and pseudotime within a branch, we used the following model (Fig. S1C):

Cell-TraitScore~Branch:Pseudotime

Note that this model differs from the interaction model used for testing branch dependency, which includes both pseudotime and the interaction term, and therefore, does not have a coefficient that directly indicates the direction of the correlation.

### 4.4 Identification of biological processes and genes underlying trait-trajectory associations

To identify biological processes that underlie the connection between trait and trajectory, we first created a ranked list for the genes in the dataset, according to the incremental change in their expression along the trajectory. We estimated these incremental changes using Monocle 3′s *fit_models* function [61], which uses GLM to model the effect of pseudotime on genes' expression (Fig. 1E). We used log-normalized gene expression values and a Gaussian error distribution GLM. The number of genes detected in each cell was added as a covariate. The t-statistics of the pseudotime coefficients were used as the scores for ranking the genes. To rank genes according to a certain branch, we replaced the pseudotime term with a term for the interaction between pseudotime and branch. The genes were sorted from lowest (reflecting declining expression along the trajectory) to highest (reflecting increasing expression along the trajectory). We then used gene-set enrichment analysis (GSEA) to identify gene sets whose genes are over-represented at the end of the list (Fig. 1F). We ran GSEA implemented in the clusterProfile R package [62], using the ontology gene set (GO, C5, 50 < set size < 500) from MSigDB [63]. Gene sets with FDR q-value < 0.05 and normalized enrichment score (NES) < 0 (indicating increased expression along the trajectory) were considered linked with the trajectory.

To identify trajectory-linked gene sets that are associated with a given trait, we used MAGMA's gene-set analysis. We used the leading-edge subset defined by GSEA as the input gene set for MAGMA's test (Fig. 1G). We used the leading-edge set rather than the whole gene set, to establish that the set of genes contributing to the link between the biological process and the trajectory was also associated with the trait. Gene sets with p-value < 0.05 were considered significant.

To prioritize genes that may carry the trajectory-trait-biological process associations, we considered the genes from the leading-edge of trajectory-linked sets that were significantly associated with the trait (MAGMA's gene-level p-value < 0.05).

## 5 Code availability

R scripts and sample input files for this pipeline are available at https://github.com/ElkonLab/scGWAS.

## 6 Author statement

The authors have seen and approved the final version of the manuscript being submitted. They warrant that the article is the authors' original work, has not received prior publication, and isn't under consideration for publication elsewhere.

## Funding

This study was supported by Israel Science Foundation (ISF) grant no. 2118/19 and DIP German–Israeli Project cooperation grant (DFG RE 4193/1-1). R.E. is a Faculty Fellow of the Edmond J. Safra Center for Bioinformatics at Tel Aviv University. ED. S was partially supported by a fellowship from the Edmond J. Safra Center for Bioinformatics at Tel Aviv University, and by Teva Pharmaceutical Industries Ltd as part of the Israeli National Forum for BioInnovators (NFBI).

## Declaration of Competing Interest

The authors declare that they have no known competing financial interests or personal relationships that could have appeared to influence the work reported in this paper.
