# Supplementary material for: Genetic mapping of developmental trajectories for complex traits and diseases
Source: Comput Struct Biotechnol J. 2021 Jun 6;19:3458–69. doi: 10.1016/j.csbj.2021.05.055 (PMC8220172; doi:10.1016/j.csbj.2021.05.055)
Supplement: Supplementary data 1 [file mmc1.docx]

**Supplemental Figure legends**

**Figure S1. A.** Illustration of a trajectory with a branching point, progenitor cells (grey) initially follow the same path until a point in which they continue their development along mutually exclusive routes, leading to the formation of either cell-type A or cell-type B. **B.** Cells are colored according to their association with the trait. Association specifically increases along the branch leading to cell-type B fate. **C.** Modeling cell-trait association using the pseudotime:branch interaction term shows that branch B is associated with the trait, while branch A is not.

**Figure S2. A.** The developmental trajectory of microglia from human fetuses (w9-18 of gestation) is most strongly associated with the risk of Alzheimer's disease. Panels are as in **Fig. 2**. **B.** Adipogenesis is associated with extreme BMI risk. Cells were sequenced from epididymal white adipose tissue of adult mice. **C**. A trajectory of nephron epithelium formation. LDL cholesterol and chronic kidney disease are strongly associated with this trajectory. **D**. Microglial activation trajectory is strongly associated with risk for multiple sclerosis.

**Figure S3. A.** Analysis of pancreatic cells from embryonic mice (E13.5 - E15.5). **B.** Progenitors differentiate into either alpha or beta cells. **C.** Cells are colored according to their association with risk for type 2 diabetes. **D.** Bar plot showing association of the pancreas trajectory with each trait. Type-2 diabetes is the most strongly associated trait. **E.** Risk for type-2 diabetes shows a significant branch dependency. **F.** Bar plots showing trait association for each of the branches. Asterisks indicate that the association shows a significant branch dependency (FDR q-value of likelihood ratio test < 0.05). **G.** Neuronal progenitors from the cortical lob and pons of human embryos and fetuses develop into either intermediate progenitor cells (IPC), or radial glial (RG) cells. **H.** The branch leading to RG cells shows a marked association with schizophrenia. **I.** Schizophrenia is the top scoring trait associated with this branch. **J.** UMAP of B Cells maturation trajectory from adult humans. Following the original publication [34], trajectory analysis was done here using Monocle 3 (rather than Monocle 2 which was used for most other datasets analyzed in our study). The map shows that progenitors develop either along a branch that forms naïve and memory B cells or a branch that forms plasma B cells. **K**. The naïve/memory branch is most associated with systemic lupus erythematosus (SLE) risk. **L.** Trajectory of cells from human fetal kidney w16 of gestation depicting the development of pretubular aggregate (PTA) cells. This differentiation trajectory involves two branching points: first, some of the cells develop along a branch that terminates in distal tubule/loop of Henle cells (DTLH), while other cells continue to develop and subsequently bifurcate into a branch that terminates in early proximal tubule (ErPrT) cells and a branch that terminates in podocyte (Pod) cells. **M.** Association with Albuminuria levels and HDL cholesterol increases along the trajectory. While association with HDL is branch dependent (significantly stronger for the podocyte branch), association with albuminuria levels is more uniform over the branches.

**Figure S4.** Same analysis as presented in Fig. 4D, shown here for additional trajectory-trait links.

**Figure S5.**  Lipoprotein traits are connected to liver and kidney developments through different biological processes.

**Figure S6.** Selected candidate genes that carry the links between traits, trajectory kinetics and enriched biological processes.

**Figure S7.** To detect association between traits and intermediate developmental states, we fitted a natural spline model (instead of a linear model). Shown are results for the following trajectory-trait connections: **A**. cortical interneurons – schizophrenia; **B**. liver – HDL levels; **C**. neuron progenitors – schizophrenia; **D**. pancreas (E14.5) – diabetes type 2; **E.** B cells – Multiple sclerosis; and **F**. B cells – SLE. In each plot, the curve fitted using the linear model used throughout the paper is shown on the left, and the curve fitted using the spline approach on the right. P-values for the spline model for none-branched trajectories (**A** and **B**) were calculated by comparing the models with and without pseudotime as an explanatory variable using VGAM's likelihood ratio testing. For the branched trajectories (**C** – **F**), we used the same test for branch-dependency as for the linear models described in Methods (using VGAM's likelihood ratio testing). **F.** Spline curve fitted to the association between the B cell naïve/memory maturation branch and SLE indicates that this association peaks at an intermediate cell state. **G.** To identify biological processes that underlie non-linear associations between traits and trajectories, we scored genes by regressing their expression against cell-trait association scores predicted by spline. Shown are the top biological processes that underlie the (non-linear) link between B cells maturation and SLE. **H**. Top candidate genes driving the link between biological processes identified as being linked to the association between B cells and SLE (that is, those identified in **G**). Note that the expression of all these genes peaks at the intermediate state of naïve B cells (mirroring the pattern shown by the cell-trait association scores along the maturation trajectory).

**Figure S8.** Results are robust over a large range of filtering cutoff values. Varying the cutoff for filtering out lowly expressed genes bears little impact on results. For each of the liver (**A**), Adipogenesis (**B**), and kidney (nephrons) (**C**) datasets, we varied the cutoff for filtering out lowly expressed genes. Using either all genes, all genes expressed in a least 10 cells (the cutoff used in our analysis), the top 50%, and 25% most expressed genes (according to the number of cells in which they were detected) yielded similar results. In contrast, analyzing the most highly variable genes (HVGs) shows a more significant impact on results. **D**. HDL association scores were calculated for liver cells using each of the gene selection criteria as in **A**. Shown are the Pearson correlation coefficients between the cell-trait association scores obtained using the different gene selection criteria. Similar analysis is shown for adipogenesis – extreme BMI (**E**), and kidney – kidney diseases (**F**).

**Figure S9.** Different trajectory inference methods applied to the liver dataset (Figure 2F) yielded similar results. **A.** Trajectory analysis with each of the six trajectory inference methods (Monocle 2 (used by the original study), Monocle 3, PAGA, SCORPIUS, Slingshot, DPT). Cells are colored according to the pseudotime inferred by the method (left), and risk scores for HDL (right). **B.** Bar plot showing the significance level (in -log10) for the association between the liver trajectory and traits for the different trajectory inference methods.

**Supplemental Tables**

**Table S1**: GWAS and single-cell RNA-seq datasets analyzed in this study

**Table S2**: Trajectory-trait associations

**Table S3**: Gene set underlying trajectory-trait associations detected by our analyses.

**Table S4**: Candidate genes that underlie trajectory-pathway-trait connections detected by our analyses.

**Supplemental Figures**


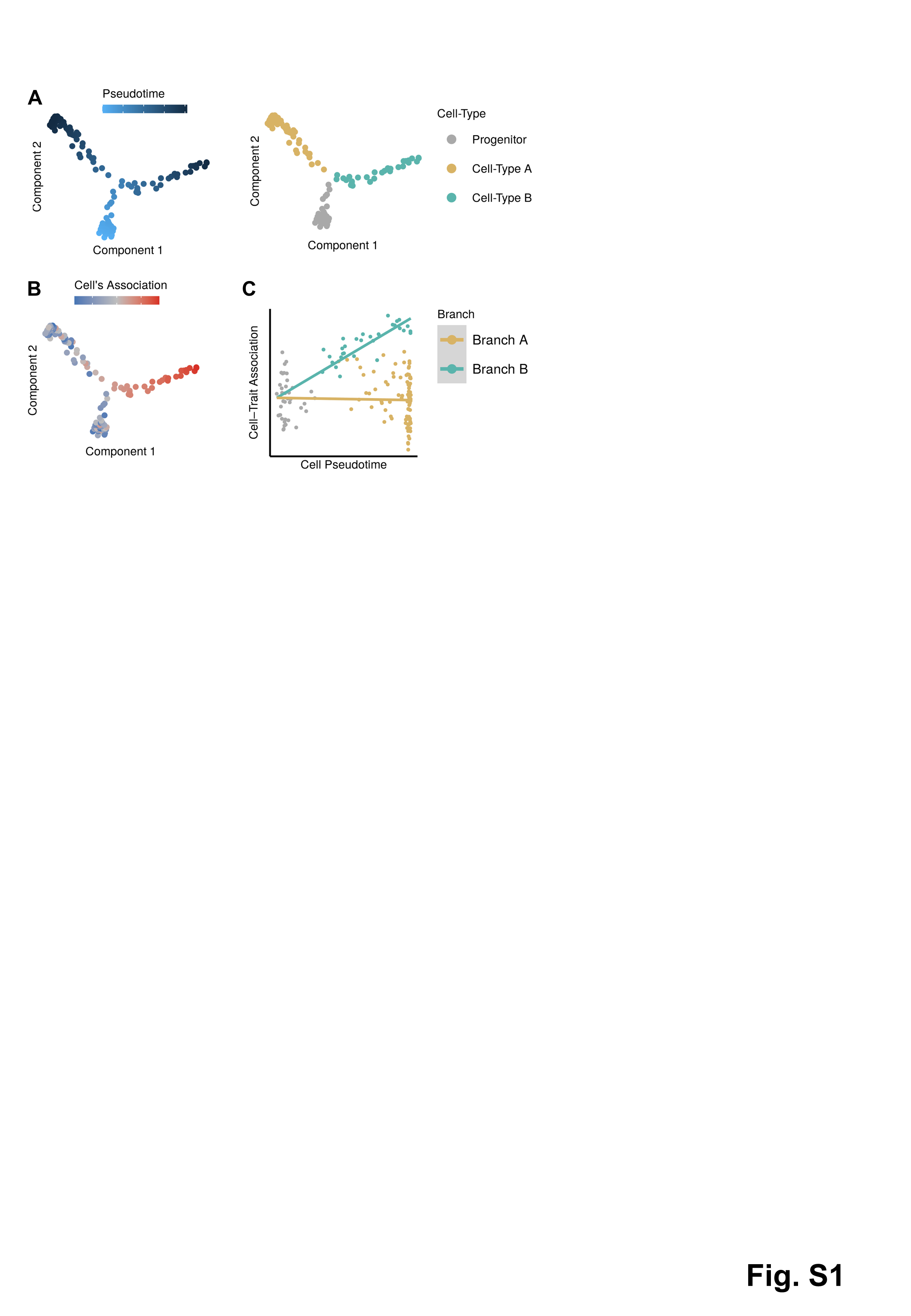


**Figure S1**


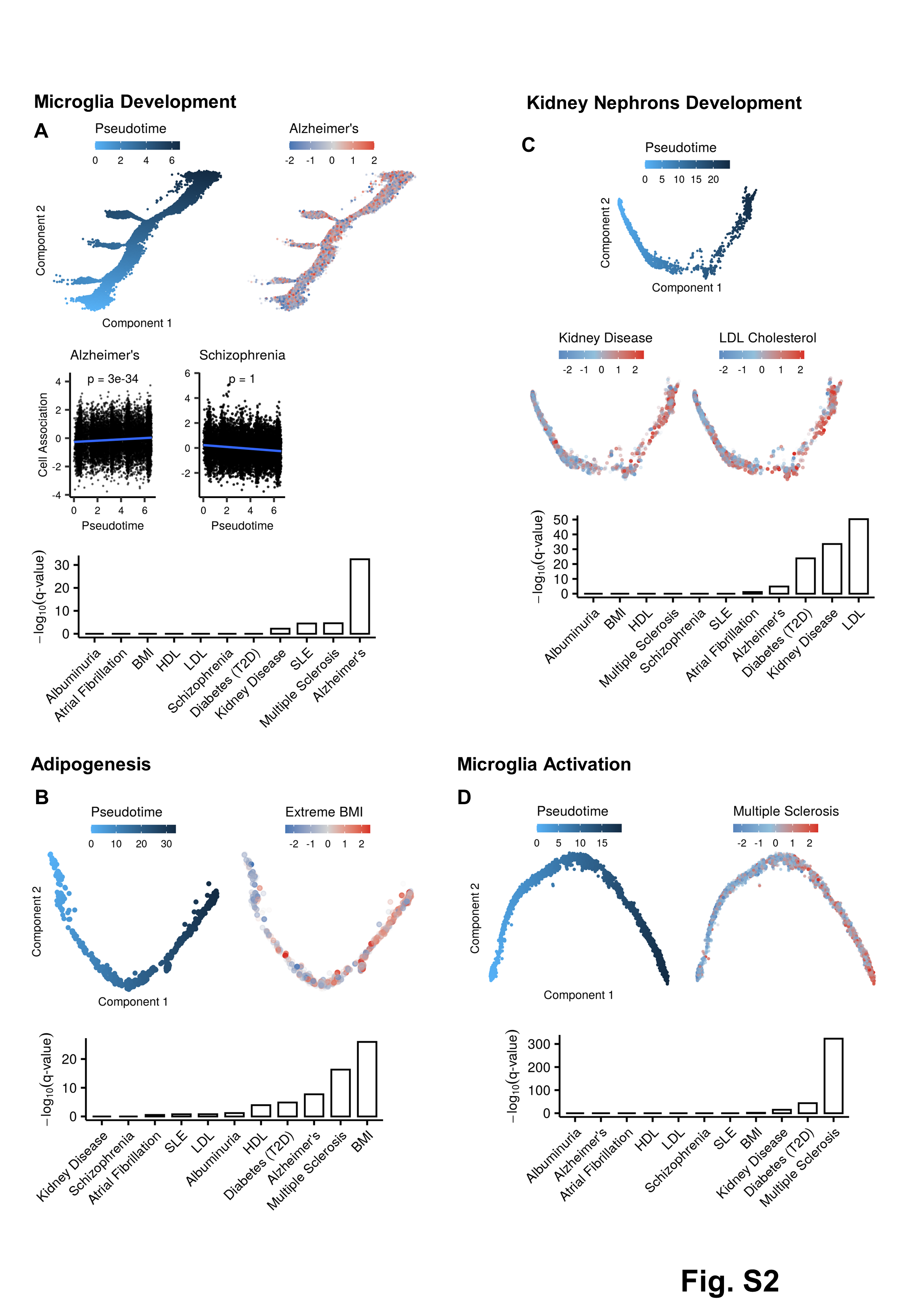


**Figure S2**


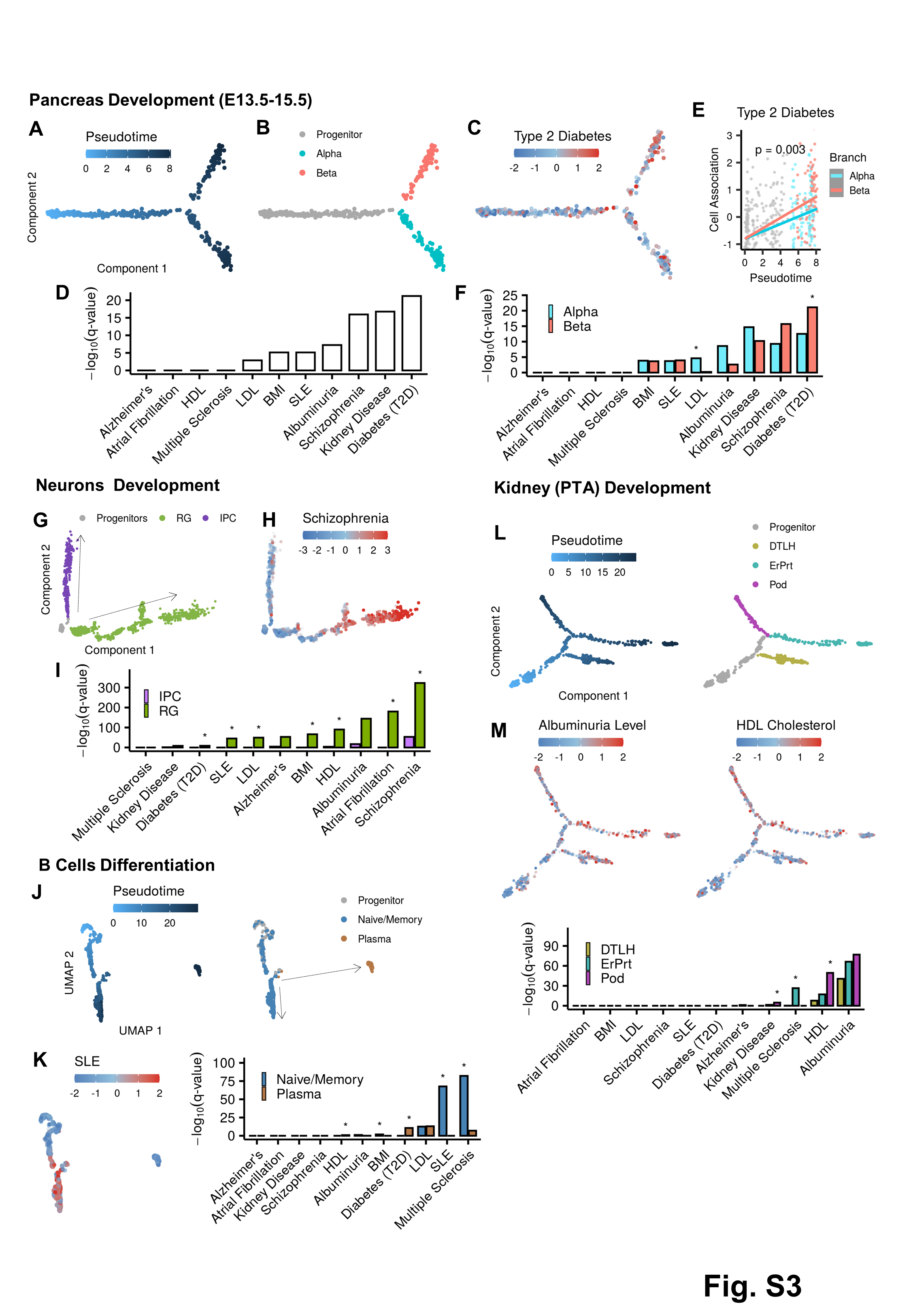


**Figure S3**


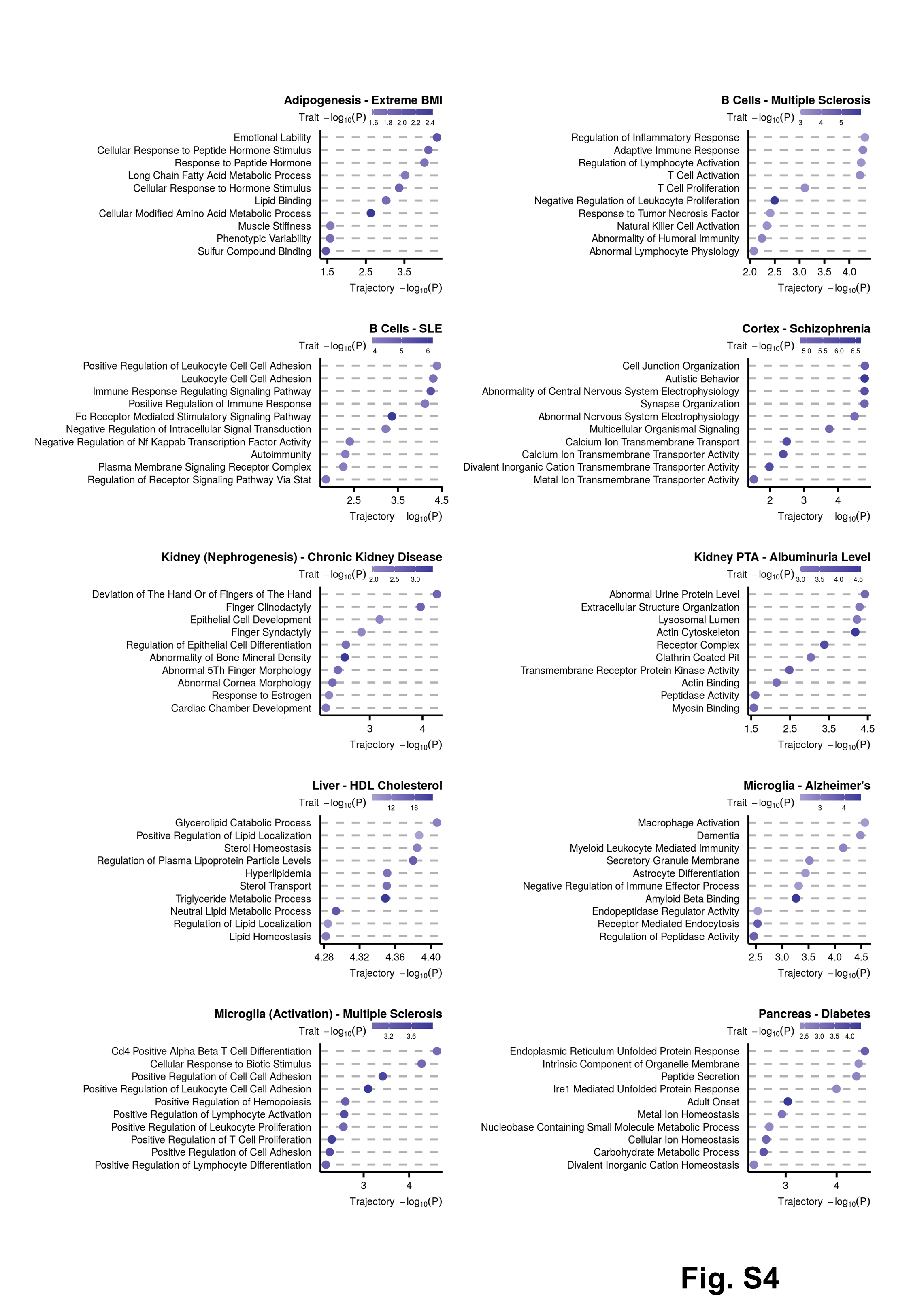


**Figure S4**

**
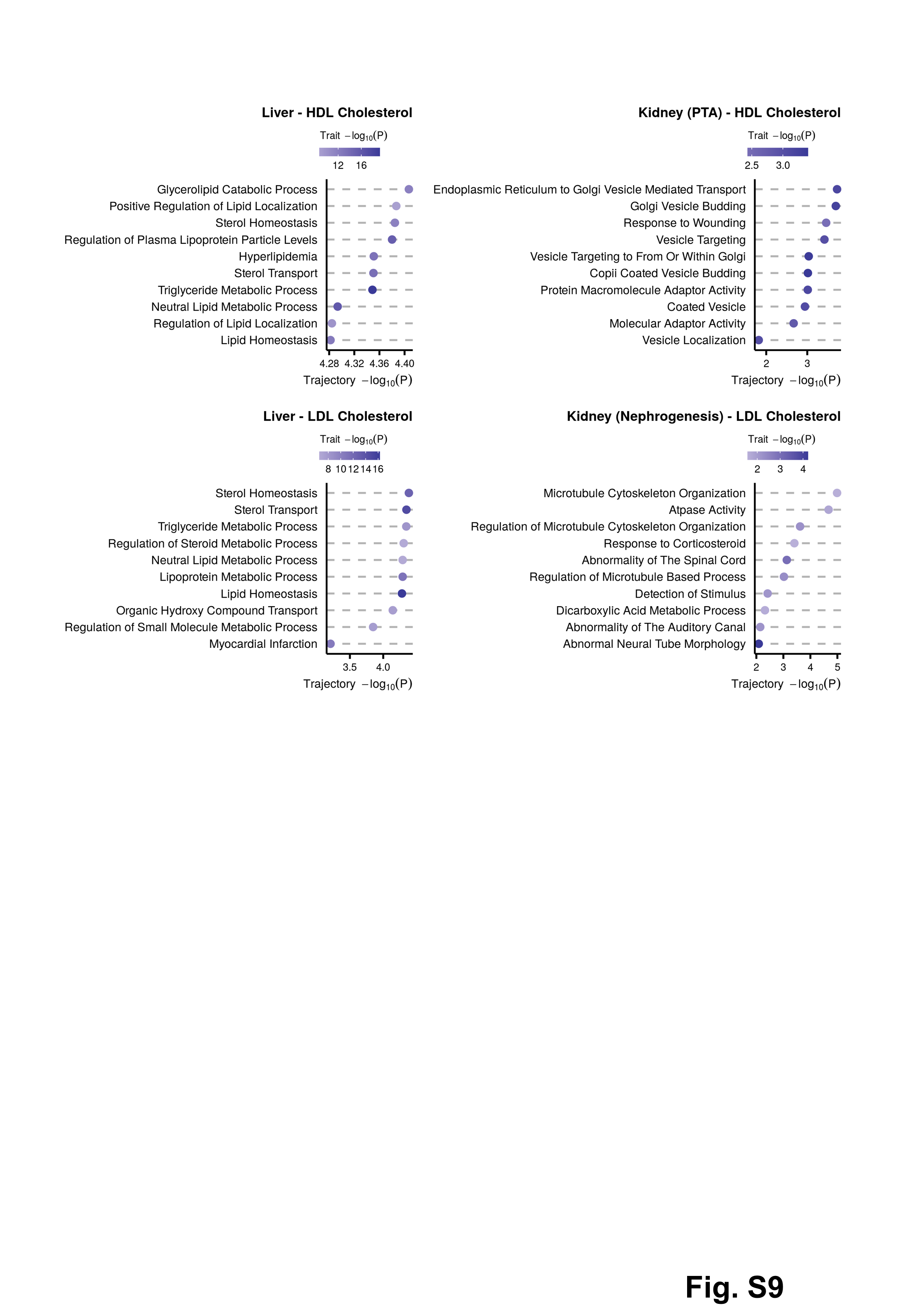
**

**Figure S5**


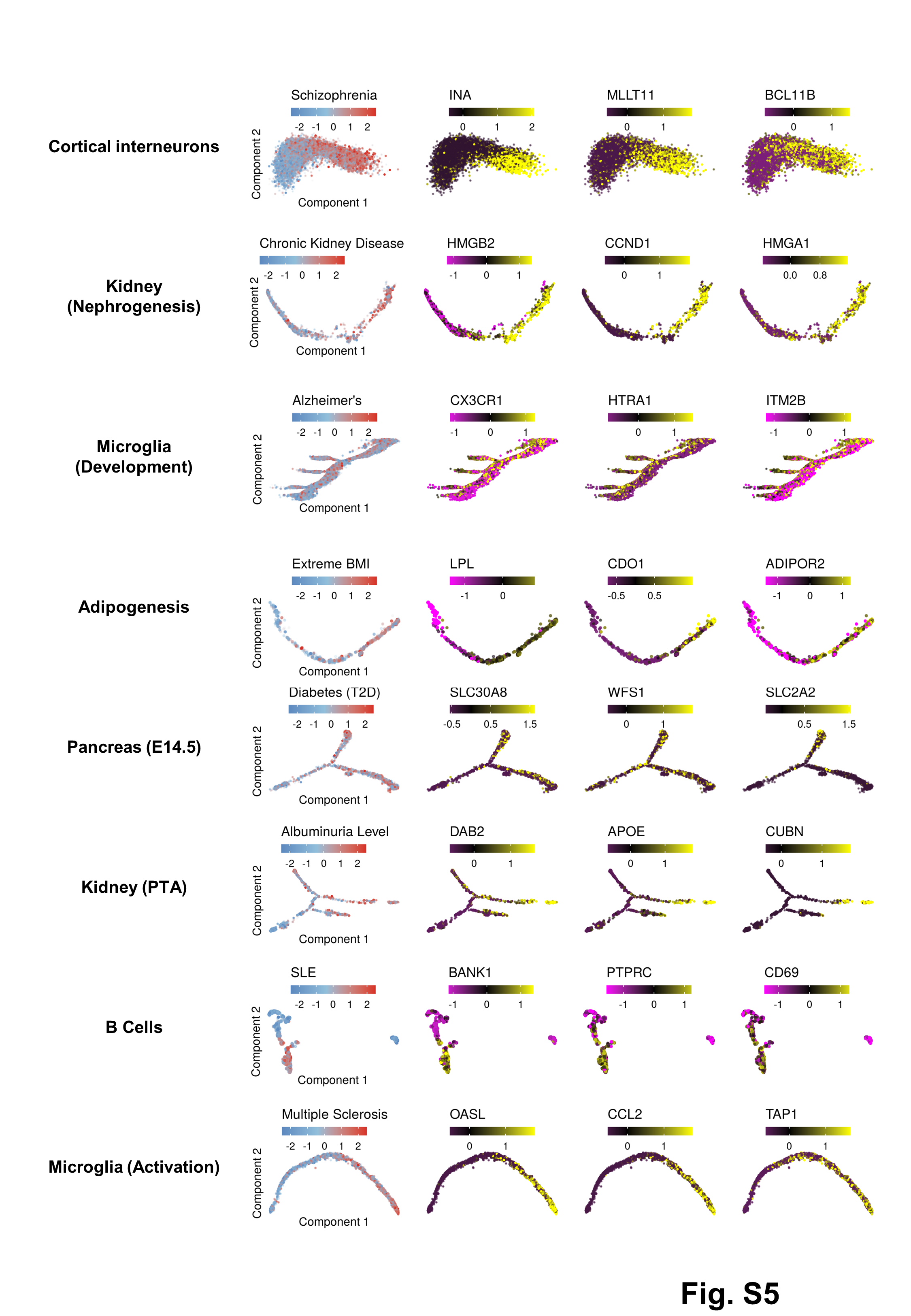


**Figure S6**


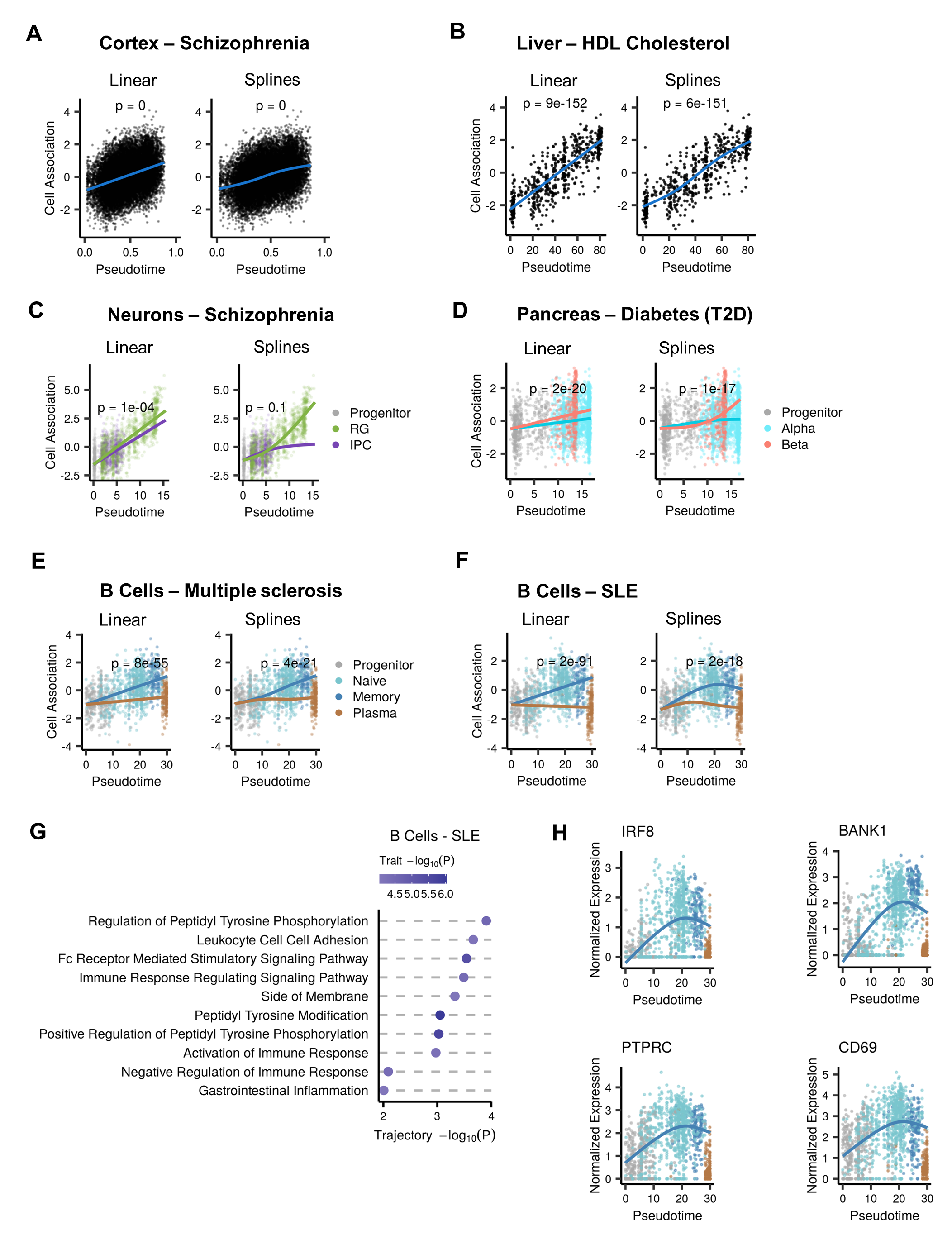


**Figure S7**

**
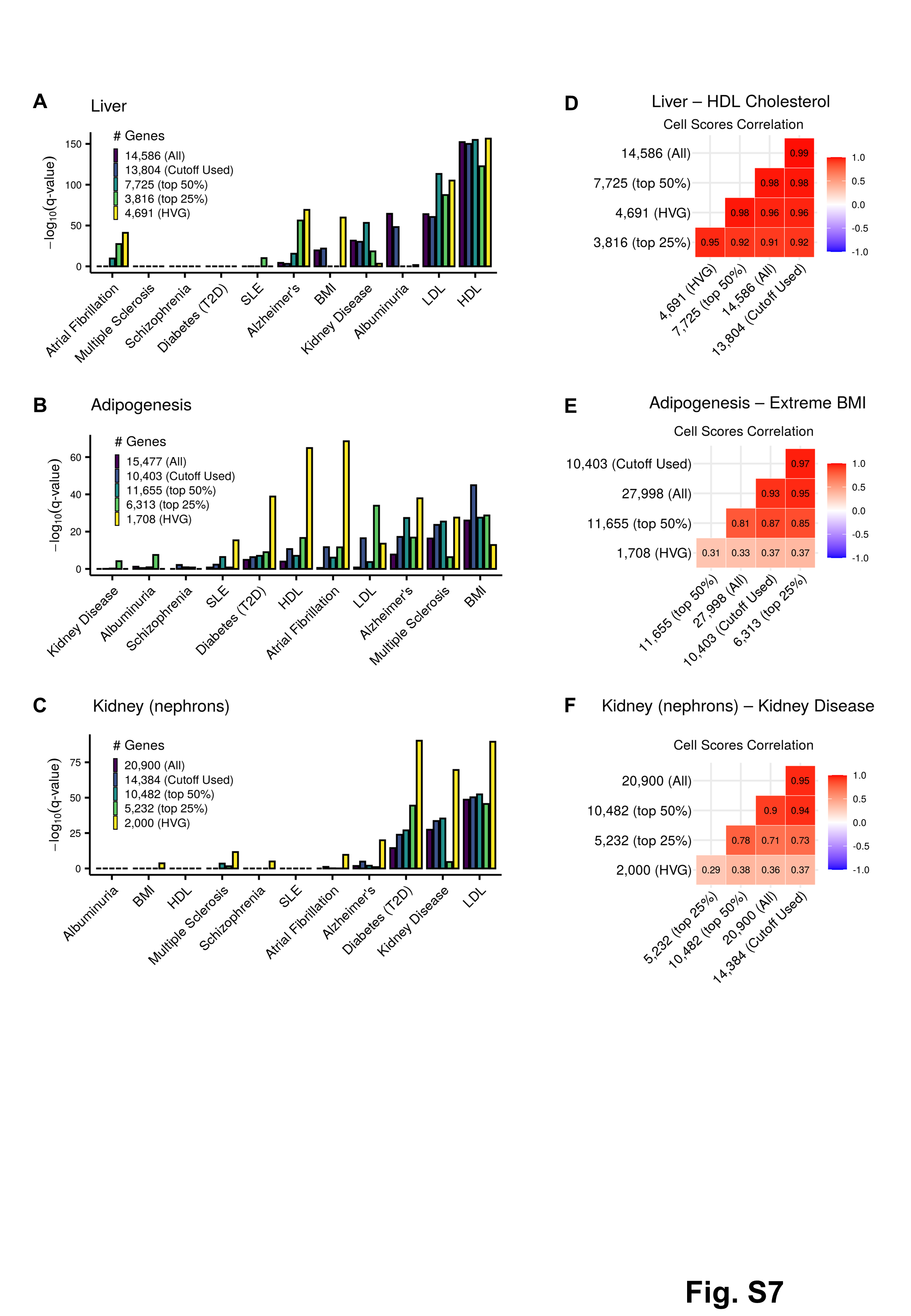
**

**Figure S8**


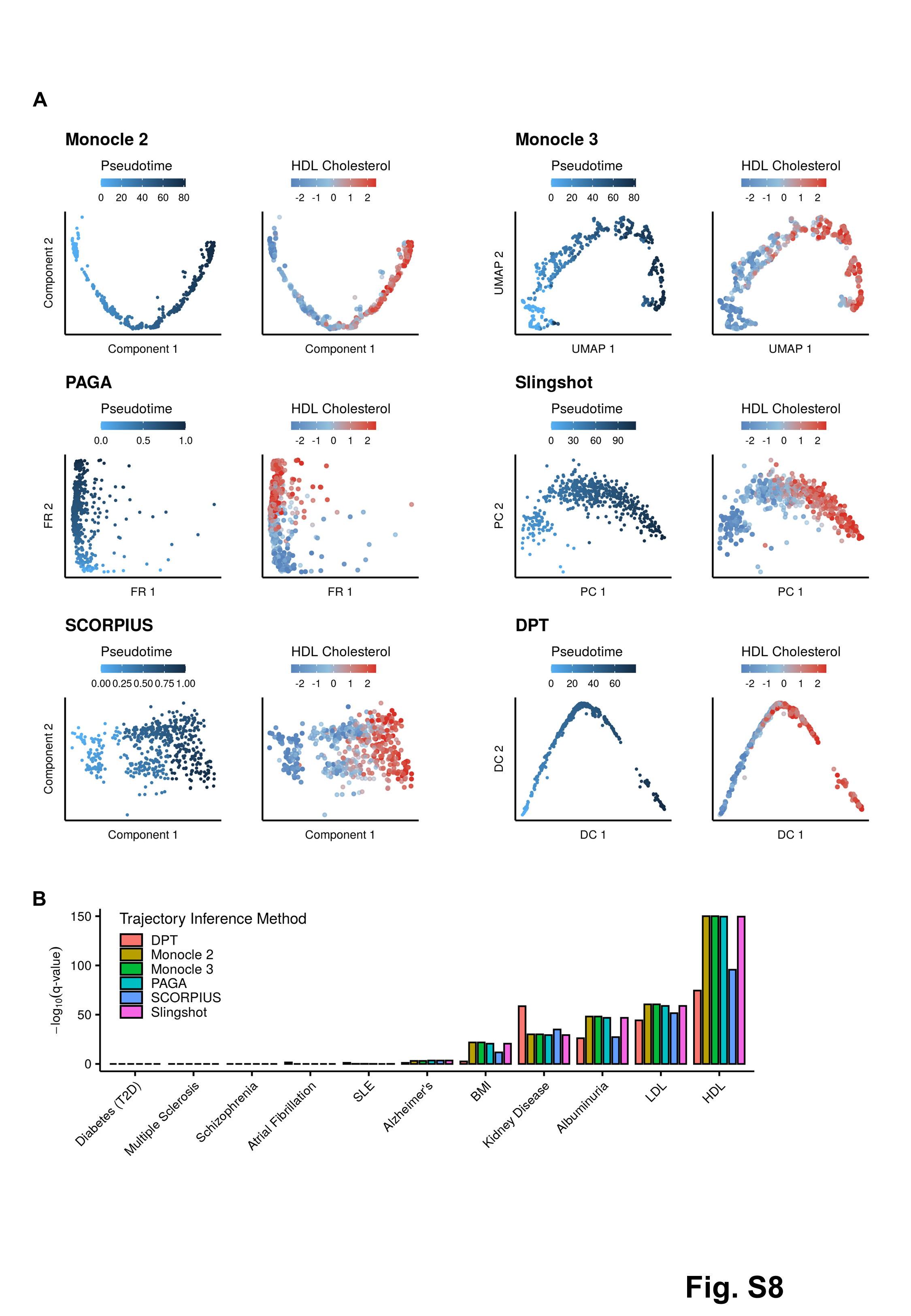


**Figure S9**
